# Increased cortical porosity and reduced cortical thickness of the proximal femur are associated with nonvertebral fracture independent of Fracture Risk Assessment Tool and Garvan estimates in postmenopausal women

**DOI:** 10.1371/journal.pone.0185363

**Published:** 2017-09-25

**Authors:** Rita Kral, Marit Osima, Tove T. Borgen, Roald Vestgaard, Elin Richardsen, Åshild Bjørnerem

**Affiliations:** 1 Department of Obstetrics and Gynaecology, University Hospital of North Norway, Tromsø, Norway; 2 Department of Clinical Medicine, UiT The Arctic University of Norway, Tromsø, Norway; 3 Department of Community Medicine, UiT The Arctic University of Norway, Tromsø, Norway; 4 Department of Orthopaedic Surgery, University Hospital of North Norway, Tromsø, Norway; 5 Department of Rheumatology, Vestre Viken Hospital Trust, Hospital of Drammen, Drammen, Norway; 6 Division of Internal Medicine, University Hospital of North Norway, Tromsø, Norway; 7 Department of Medical Biology, UiT The Arctic University of Norway, Tromsø, Norway; 8 Department of Pathology, University Hospital of North Norway, Tromsø, Norway; University of Notre Dame, UNITED STATES

## Abstract

The Fracture Risk Assessment Tool (FRAX) and Garvan Calculator have improved the individual prediction of fracture risk. However, additional bone measurements that might enhance the predictive ability of these tools are the subject of research. There is increasing interest in cortical parameters, especially cortical porosity. Neither FRAX nor Garvan include measurements of cortical architecture, important for bone strength, and providing independent information beyond the conventional approaches. We tested the hypothesis that cortical parameters are associated with fracture risk, independent of FRAX and Garvan estimates. This nested case-control study included 211 postmenopausal women aged 54–94 years with nonvertebral fractures, and 232 controls from the Tromsø Study in Norway. We assessed FRAX and Garvan 10-year risk estimates for fragility fracture, and quantified femoral subtrochanteric cortical porosity, thickness, and area from computed tomography images using StrAx1.0 software. Per standard deviation higher cortical porosity, thinner cortices, and smaller cortical area, the odds ratio (95% confidence interval) for fracture was 1.71 (1.38–2.11), 1.79 (1.44–2.23), and 1.52 (1.19–1.95), respectively. Cortical porosity and thickness, but not area, remained associated with fracture when adjusted for FRAX and Garvan estimates. Adding cortical porosity and thickness to FRAX or Garvan resulted in greater area under the receiver operating characteristic curves. When using cortical porosity (>80^th^ percentile) or cortical thickness (<20^th^ percentile) combined with FRAX (threshold >20%), 45.5% and 42.7% of fracture cases were identified, respectively. Using the same cutoffs for cortical porosity or thickness combined with Garvan (threshold >25%), 51.2% and 48.3% were identified, respectively. Specificity for all combinations ranged from 81.0–83.6%. Measurement of cortical porosity or thickness identified 20.4% and 17.5% additional fracture cases that, were unidentified using FRAX alone, and 16.6% and 13.7% fracture cases unidentified using Garvan alone. In conclusion, cortical parameters may help to improve identification of women at risk for fracture.

## Introduction

Fragility fracture is a growing health problem due to a longer lifespan and an aging population [1,2]. Therefore, it is important to identify individuals at high fracture risk, and offer them appropriate care and treatment. The most widely used measurement to assess fracture risk is areal bone mineral density (aBMD) [3–7]. However, aBMD alone has low sensitivity for fracture [3], as most of the fragility fractures occur in individuals with an aBMD in the osteopenic or normal range, and not in those with an aBMD below the osteoporosis threshold [4]. In order to address this lack of sensitivity, tools such as the Fracture Risk Assessment Tool (FRAX) [5,6] and the Garvan Fracture Risk Calculator have been developed [7].

The FRAX tool is widely used to calculate the 10-year probability of hip and major osteoporotic fracture (hip, proximal humerus, wrist, and clinical spine) based on the individual’s risk factor profile [5,6]. FRAX includes age, sex, body mass index (BMI) computed from height and weight, and clinical risk factors such as a prior fragility fracture, parental history of hip fracture, current smoking, alcohol consumption, oral glucocorticoid use, rheumatoid arthritis, other causes of secondary osteoporosis, and femoral neck (FN) aBMD. The Garvan Fracture Risk Calculator is a simpler tool [7,8], and only includes five risk factors: age, sex, number of fractures since an age of 50 years, number of falls over the last 12 months, and FN aBMD (or body weight). Garvan estimates the individual’s 5-year and 10-year absolute risk for hip fracture and any fragility fracture (hip, humerus, wrist, metacarpal, scapula, clavicle, sternum, pelvis, distal femur, proximal tibia, patella, spine [symptomatic]) [7]. Both FRAX and Garvan tools can be used with or without FN aBMD.

Additional skeletal determinants of bone strength are subject to clinical research, which may modify or enhance the predictive ability of existing tools. The FRAX estimates can be adjusted for trabecular bone score (TBS), which is an index of trabecular microarchitecture [9,10]. However, both trabecular and cortical architecture are important for bone strength [9–12], but neither FRAX nor Garvan take cortical bone architecture into account, which is particularly important for bone strength as 80% of the skeleton consists of cortical bone [11,12]. There is increasing interest in measurements of cortical parameters, which may provide independent information regarding skeletal strength and fracture risk beyond these conventional approaches.

In a prospective study, cortical area and cortical bone mass of the distal tibia, but not cortical porosity, were associated with incident fractures, independent of FN aBMD and FRAX score, in older men [13]. In contrast, reports from cross-sectional studies have suggested that cortical porosity is associated with prevalent fracture in women and men [12,14–16]. Women with fractures have higher cortical porosity and thinner cortices than controls as shown in biopsies [17] and computed tomography (CT) scans of the proximal femur [14,18,19]. Moreover, cortical porosity is associated with fracture, independent of FRAX [12,18].

Although a measurement of cortical porosity combined with FRAX identified additional women with fracture than using FRAX alone, more than half of the fracture cases were still not identified using either FRAX or cortical porosity [14]. Improving identification of individuals at high fracture risk is still a challenge. To the best of our knowledge, there is no study of the performance of cortical parameters independent of Garvan estimates. We aimed to explore this further by including cortical thickness and cortical area in the current analysis, and test whether combinations of cortical parameters with FRAX or Garvan estimates can provide additional information and improve identification of women with fracture beyond the existing tools. Therefore, this study tested the hypothesis that measurements of cortical parameters (porosity, thickness, and area) are associated with fracture risk, independent of FRAX or Garvan estimates.

## Subjects and methods

### Subjects

The Tromsø Study is a single-center, population-based study in Northern Norway, which conducted six surveys between 1974 and 2008 [20]. During the Tromsø 4 survey in 1994–95, 37,558 eligible inhabitants in Tromsø over 24 years old were invited to participate, and 27,158 (72%) agreed. Within these participants, all nonvertebral fractures that occurred between January 1, 1994 and January 1, 2010 were registered from the University Hospital of North Norway, Tromsø x-ray archives [21]. Participants with a vertebral fracture were not included in this x-ray-based fracture registry, as few of them came to the hospital for an x-ray.

In 2011, we designed a nested case–control study and identified 1,250 women from the x-ray-based fracture registry that suffered at least one fracture of the hip, wrist, or proximal humerus after the age of 50 years [14,18,19]. We invited all 760 women who were still alive and living in Tromsø. All women who were willing to participate had a pre-screening phone call to determine whether they were eligible for participation in accordance with the inclusion and exclusion criteria. Those who were premenopausal, received bisphosphonates, or had hip prostheses or metal screws in the hip region were excluded the study. Since metal on one side of the hip can create noise in the CT images on both sides, many women with a hip fracture could not be included unless they had the metal removed. After screening, 264 fracture cases were included in this study [14,18,19]. Age-matched, fracture-free women, who were within the same 5-year age group, were randomly selected from the Tromsø 4 participants and 1186 were invited. After a pre-screening phone call to determine whether they were eligible and fracture-free, 260 controls were included. Of the total 524 participants, we excluded 15 women who were currently receiving hormone replacement therapy and 66 women due to movement artifacts during CT scanning. This resulted in 443 women in the final analyses: 232 controls and 211 fracture cases (4 hip, 181 wrist, and 26 proximal humerus). The median time since their index fracture was 6.6 y (range: 1–25 y). All variables included in the analysis were based on information obtained at the time of study enrollment between November 2011 and January 2013. All participants provided written informed consent. The study was approved by the Regional Committee for Medical and Health Research Ethics (REK Sør-Øst) (reference 2010/2282) and was conducted in accordance with the World Medical Association Declaration of Helsinki.

### Methods

#### Variables and measurements

At enrollment of the study, the participants filled in a questionnaire that included information concerning all fractures after the age of 50 years (number and type of fracture), number of falls in the last year, diseases, use of medication, and lifestyle. Height and weight were measured while wearing light clothing and without shoes. BMI was calculated as weight/height^2^. FN aBMD was measured using dual-energy x-ray absorptiometry (GE Lunar Prodigy, Lunar Corporation, Madison, WI, USA) and the coefficient of variation was 1.7%.

We entered the data collected at enrollment into the online country-specific FRAX algorithm for Norway to calculate the individual 10-year probability of a major osteoporotic fracture (http://www.shef.ac.uk/FRAX/), and the Garvan Fracture Risk Calculator to calculate the 10-year fracture risk for any fragility fracture (http://garvan.org.au/promotions/bone-fracture-risk/calculator/). An age of 90 years was used to obtain FRAX estimates in individuals older than 90 years of age. We included FN aBMD in the calculation of FRAX and Garvan estimates. The index fractures used as the inclusion criteria for this study were not included as a “previous fracture” in the calculation of the FRAX estimate, because the aim was to assess 10-year probability of fracture before the event, not the probability of fracture after this event [12, 14]. The index fractures were not included in the number of fractures in the Garvan estimate.

However, the “previous fractures” (before the index fracture) and “subsequent fractures” (after the index fracture) should both be used equally in the calculation of FRAX and Garvan estimates. Therefore, we validated these fractures through the medical records of 91 women, who either had a self-reported “previous fracture” (n = 54), a total of two or more self-reported fractures (n = 71), or both (n = 34). The validation confirmed that 61 of 91 women had a previous or subsequent fracture, which we included in the calculation of their FRAX estimates. The same 61 women had one or more fractures, which we included in the calculation of their Garvan estimates.

CT scans (Siemens Somatom Sensation 16, Erlangen, Germany) of the non-dominant hip were performed at the Department of Radiology in the University Hospital of North Norway [14]. The CT machine had an in-plane resolution of 0.74 mm and the slice thickness was set at 0.6 mm. The hip was scanned from just above the femoral head to 2 cm below the lesser trochanter, and the exposure dose of radiation was ~1.5 mSv [14]. CT scans of the hip were performed at 120 kV, with a pitch of 0.75, using 90 mA, and reconstructed using a fixed field of view at 120 mm [22]. Quality control was carried out by scanning a phantom containing rods of hydroxyapatite (QRM Quality Assurance in Radiology and Medicine GmbH, Moehrendorf, Germany). The CT images were sent to Melbourne, Australia, and analyzed by collaborators, who were blinded to the fracture status, using the StrAx1.0 software (StraxCorp Pty Ltd, Melbourne, Australia). As cortices are thin at the most proximal femur (femoral head, neck, and trochanter), analyses were confined to a 3.7 mm subtrochanteric region of interest with thicker cortices, which started at the tip of the lesser trochanter as shown previously [14,23].

The StrAx1.0 software is a non-thresholding method that automatically segments the bone within the region of interest into its compartments: compact cortex and outer and inner transitional zones (TZ) [14,23]. This was performed similarly in low-resolution images [14,23] as in high-resolution images [24]. Of the total cortex at this subtrochanteric site, 70.0% was compact cortex, while 22.3% and 11.7% were outer and inner TZ, respectively. Porosity within the total cortex and each cortical compartment was quantified automatically throughout the region of interest using the StrAx1.0 software [14,23,24] and coefficient of variation was 0.3–2.3% [14,23]. The agreement (R^2^) between CT and high-resolution peripheral quantitative computed tomography (HR-pQCT) ranged from 0.86 to 0.96 for quantification of porosity at the same femoral subtrochanteric site [14,23]. The correlation between porosity (ranged from 40 to 95%), quantified using CT and HR-pQCT, was linear [23].

The porosity quantified by this algorithm is the proportion of emptiness within each voxel or the fraction of the bone occupied by void [24, 25]. StrAx1.0 quantifies porosity in low-resolution images, and similarly for high-resolution images, even though pores are not visible. It is a density-based, indirect measure of porosity, and the size and number of pores are not determined [14,18,19,23,25]. StrAx1.0 software quantifies porosity as a fraction of void, regardless of size of the pores, and indirectly captures porosity produced by large and small pores. It accounts for partial volume effect by including not only void within completely empty voxels, but also partly empty voxels [24]. By using the StrAx1.0 software, we can quantify porosity of the compact cortex and the TZ. It is thus more inclusive than traditional measurements, and the porosity is higher than what has been previously reported using other methods [24,25].

#### Statistical analyses

Age-adjusted analysis of variance was used to compare cases and controls. Logistic regression analysis was used to calculate odds ratio (OR) for fracture with 95% confidence interval (CI) adjusted for age, height, weight, and FN aBMD, or adjusted for FRAX or Garvan estimates. Due to skewed distribution of FRAX and Garvan estimates, we used log-transformed variables in the models. To further discriminate fracture cases from controls, the area under the receiver operating characteristic curve (AUC) was obtained using logistic regression models for FRAX and Garvan estimates alone, and after adding cortical parameters (porosity, thickness, or area). Sensitivity and specificity for fracture were explored at selected thresholds for FRAX estimates above 15%, 20%, and 25%, Garvan estimates above 15%, 20%, and 25%, cortical porosity above the 75^th^, 80^th^, and 90^th^ percentile, and cortical thickness below the 10^th^, 20^th^, and 25^th^ percentile. We chose specificity above 85% as a reasonable criterion for selection of thresholds for each of the variables and for further analysis of combinations of variables. We further calculated the net reclassification improvement (NRI) to quantify how well the new models correctly reclassified women [26]. Each of the original models with FRAX alone or Garvan alone was compared with a new model, which was the original model plus cortical porosity or cortical thickness. The net proportion of women reclassified correctly were calculated from the number of women with and without events reclassified correctly or incorrectly. When we designed this study, we used EpiInfo (version 2008) for power calculation to assess the number of participants needed. With cortical porosity as a continuous variable, we chose a threshold to define who was exposed. Assuming a power of 80%, and a significance level of 5%, we would be able to detect an OR = 2.0 with 165 fracture cases and 165 controls (1:1), OR = 1.8 with 230 cases and 230 controls (1:1), and OR = 1.6 with 363 cases and 363 controls (1:1), if 25% of the sample was exposed to high porosity. Analyses were performed using SAS Software package, v9.4 (SAS Institute Inc., Cary, NC, USA) and *p* < 0.050 was considered significant.

### Results

#### FRAX, Garvan, and cortical bone parameters in cases and controls

Women with nonvertebral fracture were taller, had lower BMI, lower FN aBMD, and higher FRAX and Garvan estimates than age-matched, fracture-free controls (*p* < 0.050 for all; Table 1). Cases had higher cortical porosity, thinner cortices, and smaller cortical area at the femoral subtrochanter (*p* < 0.050 for all). There was no difference between cases and controls in self-reported health, weekly hours of physical activity, and number of falls during the last year.

**Table 1:** Characteristics of postmenopausal women by fracture status.

	Cases		Controls	p-value
n	211		232	
Age (year)	68.4 ± 7.7		68.3 ± 6.7	0.937
Height (cm)	162.7 ± 6.1		161.2 ± 6.6	0.011
Weight (kg)	68.9 ± 10.5		70.0 ± 10.8	0.280
Body mass index (kg/m^2^)	26.0 ± 3.8		27.0 ± 4.3	0.015
Self-reported good health, n (%)	147 (70.3)		165 (71.1)	0.860
Physical activity (hour/week)	2.6 ± 1.6		2.5 ± 1.7	0.421
Currently smoker, n (%)	29 (13.7)		24 (10.3)	0.257
Alcohol intake (drink/week)	3.2 ± 3.7		3.3 ± 3.5	0.407
History of previous fracture, n (%)	61 (28.9)		0	
Parental hip fracture, n (%)	34 (16.1)		37 (16.0)	0.469
Rheumatoid arthritis, n (%)	11 (5.2)		8 (3.5)	0.407
Oral glucocorticoid use, n (%)	8 (3.8)		2 (0.9)	0.023
Take calcium supplements, n (%)	44 (20.9)		28 (12.1)	0.007
Take vitamin D supplements, n (%)	163 (77.3)		166 (71.6)	0.278
Hyperthyroidism, n (%)	8 (3.8)		6 (2.6)	0.468
Hypothyroidism, n (%)	40 (19.0)		20 (8.6)	0.002
Ulcerative colitis/Crohn’s disease, n (%)	12 (5.7)		5 (2.2)	0.054
Diabetes, n (%)	9 (4.3)		13 (5.6)	0.513
Early menopause < 45 years, n (%)	34 (16.1)		22 (9.5)	0.036
eGFR (ml/min)	77.4 ± 16.8		77.8 ± 14.9	0.584
eGFR below 60 ml/min, n (%)	25 (11.9)		22 (9.5)	0.409
Femoral neck (FN) aBMD (mg/cm^2^)	794 ± 100		860 ± 110	< 0.001
FRAX estimate with FN aBMD (%)	15.2 ± 7.8		10.8 ± 4.9	< 0.001
Garvan estimate with FN aBMD (%)	22.6 ± 13.3		14.4 ± 6.5	< 0.001
Number of fracture >50 years, n (%)*				
1	44 (20.9)		0	
2	15 (7.1)		0	
≥3	2 (1.0)		0	
Number of falls in past year, n (%)				
0	138 (65.4)		147 (63.4)	
1	58 (27.5)		71 (30.6)	
2	14 (6.6)		12 (5.2)	
≥3	1 (0.5)		2 (0.9)	
Femoral subtrochanter architecture				
Total bone vBMD (mg HA/cm^3^)	684 ± 113		750 ± 90.0	< 0.001
Cortical porosity (%)	43.8 ± 4.35		41.7 ± 3.39	< 0.001
Cortical thickness (mm)	4.06 ± 0.58		4.36 ± 0.54	< 0.001
Cortical cross-sectional area (mm^2^)	409 ± 39.1		417 ± 39.4	0.029
Cortical vBMD (mg HA/cm^3^)	1025 ± 72.6		1059 ± 56.6	< 0.001
Cortical bone mineral content (mg HA)	1552 ± 184		1636 ± 174	< 0.001
Trabecular BV/TV (%)	0.266 ± 0.241		0.272 ± 0.314	0.806

Numbers are mean ± standard deviation or number (%).*Total number of fracture did not include index fractures. Cases and controls were compared using analysis of variance adjusted for age.

eGFR, estimated glomerular filtration rate; aBMD, areal bone mineral density; vBMD, volumetric BMD; HA, hydroxyapatite; BV/TV, bone volume/tissue volume; FRAX, Fracture Risk Assessment Tool for calculation of the 10-year probability of major fracture; Garvan, Fracture Risk estimate of the 10-year fracture risk for any fragility fracture.

#### FRAX, Garvan, cortical parameters, and odds for fracture

Each standard deviation higher for FRAX and Garvan estimates increased the odds for fracture; OR (95% CI) were 2.04 (1.64–2.53) and 2.31 (1.84–2.91), respectively (Table 2). Each standard deviation higher for cortical porosity, thinner cortices, and smaller cortical cross-sectional area at the femoral subtrochanter increased odds for fracture (1.71 [1.38–2.11], 1.79 [1.44–2.23], and 1.52 [1.19–1.95], respectively). We explored each component of the FRAX and Garvan tools. Early menopause and hypothyroidism were associated with increased odds for fracture, independent of age, height, and weight (1.81 [1.01–3.23] and 2.43 [1.36–4.34], respectively). Women with one or more falls within the last 12 months had no increased odds for fracture than those without falls (0.92 [0.62–1.36]).

**Table 2:** Odds ratio (OR) and 95% confidence interval (CI) for non-vertebral fracture for each of the risk factors included in FRAX or Garvan estimates, and for the femoral subtrochanter architecture.

	SD unit	OR (95% CI)	p-values
Age	+ 7.21 year	1.13 (0.92–1.39)	0.242
Height	+ 6.40 cm	1.39 (1.12–1.72)	0.003
Weight	– 10.7 kg	1.19 (0.98–1.46)	0.085
Currently smoker	yes vs no	1.41 (0.78–2.56)	0.261
Parental hip fracture	yes vs no	0.97 (0.58–1.62)	0.892
Glucocorticoid use	yes vs no	5.08 (1.03–25.2)	0.047
Rheumatoid arthritis	yes vs no	1.95 (0.75–5.06)	0.170
Hyperthyroidism	yes vs no	1.63 (0.55–4.85)	0.383
Hypothyroidism	yes vs no	2.43 (1.36–4.34)	0.003
Ulcerative colitis/Crohn’s disease	yes vs no	2.81 (0.96–1.04)	0.060
Diabetes	yes vs no	0.46 (0.08–0.77)	0.774
Early menopause < 45 year	vs ≥ 45 year	1.81 (1.01–3.23)	0.045
Femoral neck (FN) aBMD	– 0.111 mg/cm^2^	2.11 (1.66–2.68)	< 0.001
FRAX estimate (%)	+ 6.82%	2.04 (1.64–2.53)	< 0.001
Falls in the last 12 months	≥1 vs 0	0.92 (0.62–1.36)	0.675
Garvan estimate (%)	+ 12.6%	2.31 (1.84–2.91)	< 0.001
Femoral subtrochanter architecture			
Cortical porosity	+ 4.01%	1.71 (1.38–2.11)	< 0.001
Cortical thickness	– 0.58 mm	1.79 (1.44–2.23)	< 0.001
Cortical cross-sectional area	– 39.5 mm^2^	1.52 (1.19–1.95)	0.001
Cortical vBMD	– 66 mg HA/cm^3^	1.71 (1.38–2.11)	< 0.001
Cortical bone mineral content	– 183 mg HA	1.91 (1.51–2.42)	0.001

SD, standard deviation; aBMD, areal bone mineral density; vBMD, volumetric BMD; HA, hydroxyapatite; FRAX, Fracture Risk Assessment Tool for calculation of the 10-year probability of a major osteoporotic fracture; Garvan, Fracture Risk estimate of the 10-year fracture risk for any fragility fracture. Both FRAX and Garvan estimates are log-transformed and included FN aBMD.

Cortical porosity remained independently associated with fracture after adjustment for FN aBMD, FRAX, or Garvan estimates (1.39 [1.10–1.74], 1.53 [1.22–1.90] and 1.45 [1.16–1.81]) (Table 3). Cortical thickness remained independently associated with fracture after adjustment for FN aBMD, FRAX, or Garvan (1.46 [1.15–1.85], 1.47 [1.17–1.83], and 1.38 [1.10–1.73], respectively). When both cortical porosity and thickness were included in the same models with FN aBMD, FRAX, or Garvan estimates, cortical porosity remained associated with fracture, but cortical thickness did not. However, cortical cross-sectional area did not remain associated with fracture after adjustment for FN aBMD, FRAX, or Garvan estimates.

**Table 3:** Odds ratio (OR) and 95% confidence interval (CI) for nonvertebral fracture per standard deviation (SD) difference in each of cortical porosity, thickness, and cross-sectional area (CSA).

		Covariates in each of the models	OR (95% CI)
Cortical porosity	+ 4.01%	Age, height, weight, FN aBMD	1.39 (1.10–1.74)
		FRAX alone	1.53 (1.22–1.90)
		Garvan alone	1.45 (1.16–1.81)
Cortical thickness	– 0.58 mm	Age, height, weight, FN aBMD	1.46 (1.15–1.85)
		FRAX alone	1.47 (1.17–1.83)
		Garvan alone	1.38 (1.10–1.73)
Cortical CSA	– 39.5 mm^2^	Age, height, weight, FN aBMD	1.06 (0.80–1.41)
		FRAX alone	1.02 (0.83–1.26)
		Garvan alone	0.94 (0.75–1.16)

FN aBMD; femoral neck areal bone mineral density; FRAX, Fracture Risk Assessment Tool for calculation of the 10-year probability of a major osteoporotic fracture; Garvan, Fracture Risk estimate of the 10-year fracture risk for any fragility fracture.

FRAX and Garvan estimates are used log-transformed, and both estimates included FN aBMD.

#### Discrimination of fracture

**Fig 1:**
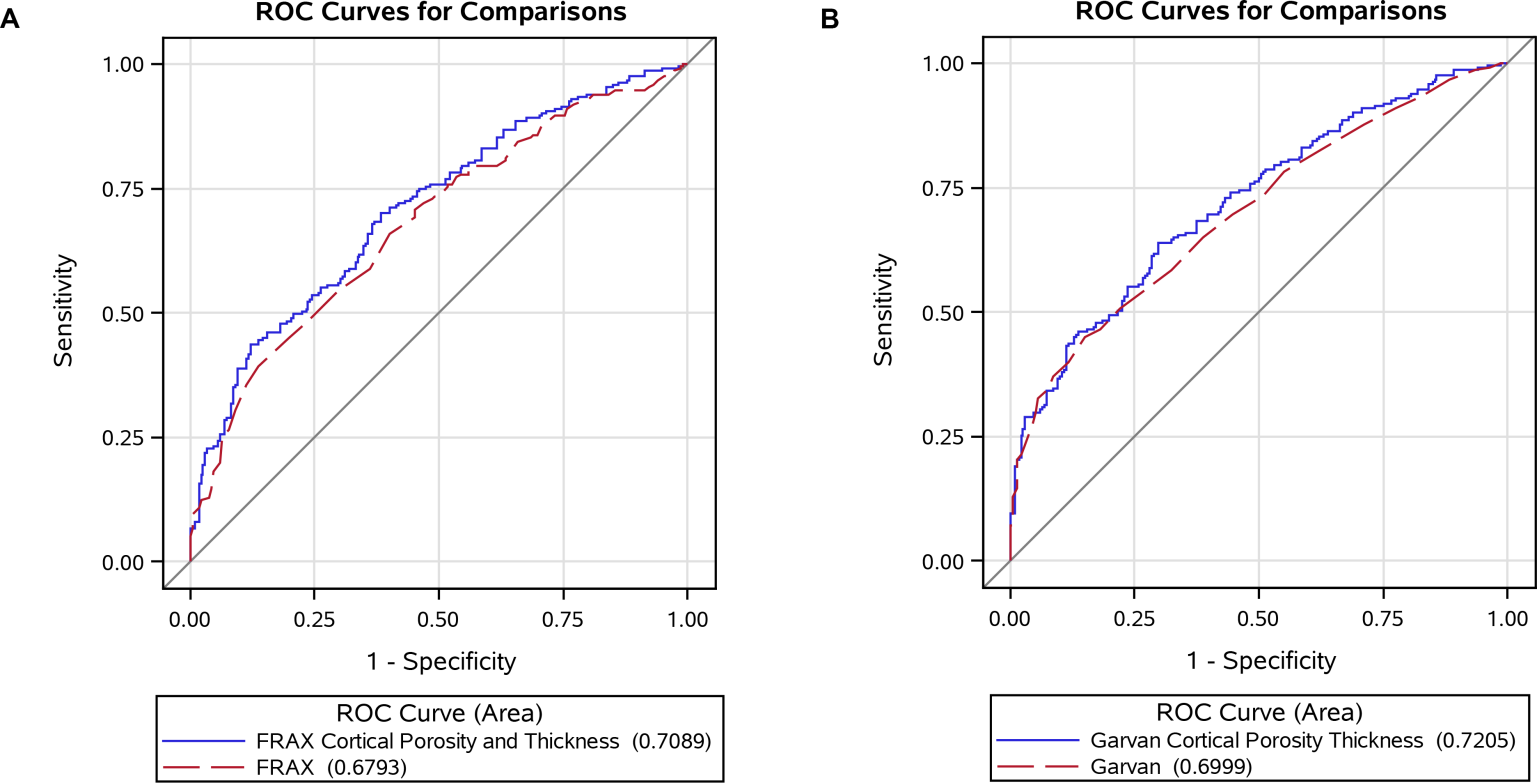
Area under the receiver operating characteristic curve (AUC) for comparison of (A) Fracture Risk Assessment Tool (FRAX) estimate before and after adding cortical porosity and thickness and (B) Garvan estimate before and after adding cortical porosity and thickness.

AUC for age and FN aBMD was 0.683, and AUC for FRAX alone was 0.679. Adding cortical porosity to FRAX improved the discrimination of fracture cases from controls over FRAX alone, and resulted in a slightly higher AUC of 0.705 (*p* = 0.051). Additionally, adding both cortical porosity and thickness to FRAX resulted in AUC of 0.709 (*p* = 0.031; Fig 1). For Garvan estimate alone AUC was 0.700, and adding both cortical porosity and thickness to the Garvan estimate resulted in a marginally higher AUC of 0.721 (*p* = 0.064).

#### Identification of fracture cases using Garvan, FRAX, and cortical bone parameters

**Fig 2:**
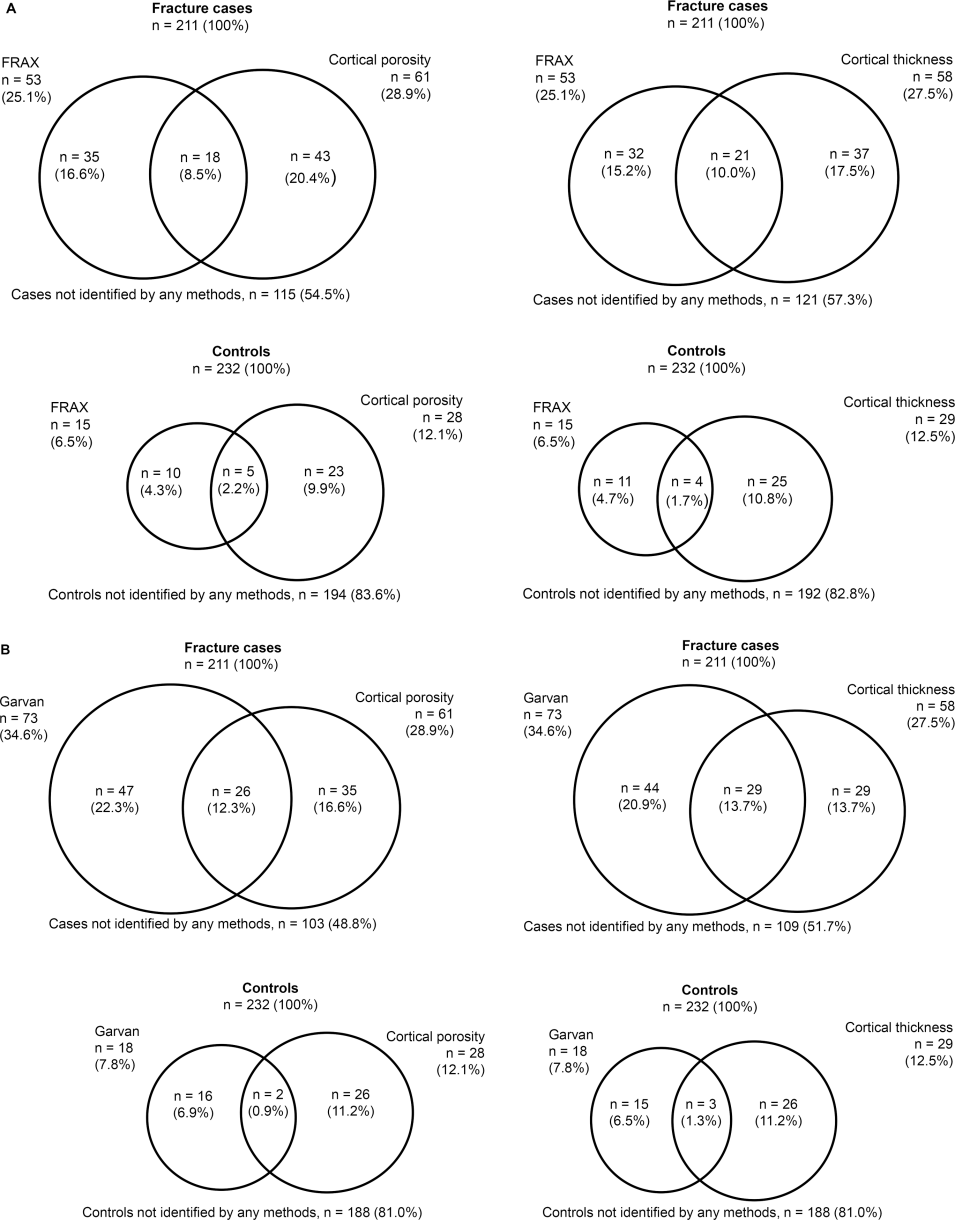
Venn diagrams show the number and proportion of woman identified using threshold for (A) Fracture Risk Assessment Tool (FRAX) estimate >20%, cortical porosity >80^th^ percentile, and cortical thickness <20^th^ percentile, and (B) Garvan estimate >25%, cortical porosity >80^th^ percentile, and cortical thickness <20^th^ percentile.

FRAX estimate (>20%) identified 25.1% of women with fracture, Garvan estimate (>25%) identified 34.6%, cortical porosity (>80^th^ percentile) identified 28.9%, and cortical thickness (<20^th^ percentile) identified 27.5% (Table 4 and Fig 2). Sensitivity at these thresholds for FRAX and Garvan estimates and cortical porosity and thickness was 25%, 35%, 29%, and 28%, respectively, and specificity was 94%, 92%, 88%, and 88%, respectively. Combining FRAX with cortical porosity and thickness identified 45.5% and 42.7% of fracture cases, respectively, and combining Garvan with cortical porosity and thickness identified 51.2% and 48.3%, respectively. Measuring cortical porosity and thickness identified additional fracture cases than using FRAX alone (20.4% and 17.5%, respectively). Additionally, measuring cortical porosity and thickness also identified additional fracture cases than using Garvan alone (16.6% and 13.7%, respectively).

**Table 4:** Sensitivity and specificity for each factor and for combinations with 95% confidence interval (CI).

	Sensitivity (%)	95% CI	Specificity (%)	95% CI
**For each factor**				
FRAX estimate >15%	45.0	38.2–52.0	80.2	74.3–85.0
FRAX estimate >20%	25.1	19.5–31.6	93.5	89.3–96.2
FRAX estimate >25%	12.3	8.35–17.7	97.8	94.8–99.2
Garvan estimate >15%	69.7	62.9–75.7	55.2	48.5–61.6
Garvan estimate >20%	46.5	39.6–53.4	81.9	76.2–86.5
Garvan estimate >25%	34.6	28.3–41.5	92.2	87.8–95.2
Cortical porosity >75^th^ percentile (>45.1%)	34.1	27.8–41.0	83.2	77.6–87.6
Cortical porosity >80^th^ percentile (>45.7%)	28.9	22.9–35.5	87.9	83.0–91.8
Cortical porosity >90^th^ percentile (>48.2%)	16.1	11.6–21.9	95.3	91.4–97.5
Cortical thickness <10^th^ percentile (<3.50 mm)	16.1	11.6–21.9	95.7	92.0–97.8
Cortical thickness <20^th^ percentile (<3.75 mm)	27.5	21.7–34.1	87.5	82.4–91.3
Cortical thickness <25^th^ percentile (<3.85 mm)	33.7	27.4–40.5	83.2	77.6–87.6
**For combinations**				
FRAX >20% or cortical porosity >80^th^ percentile	45.5	38.7–52.5	83.6	78.1–88.0
FRAX >20% or cortical thickness <20^th^ percentile	42.7	35.9–49.6	82.8	77.1–87.3
Garvan >25% or cortical porosity >80^th^ percentile	51.2	44.3–58.1	81.0	75.3–85.7
Garvan >25% or cortical thickness <20^th^ percentile	48.3	41.5–55.3	81.0	75.3–85.7
Cortical porosity >80^th^ or thickness <20^th^ percentile	39.8	33.2–46.8	79.7	73.9–84.6

FRAX: Fracture Risk Assessment Tool for calculation of the 10-year probability of major osteoporotic fracture with femoral neck areal bone mineral density (FN aBMD) included in the estimate. Garvan:10-year fracture risk estimate for any fragility fracture with FN aBMD included in the estimate.

#### Improved reclassification of fracture

After adding cortical porosity to FRAX, 43 women with fracture (20.4%) were correctly reclassified upward, 23 women without fracture (9.9%) were incorrectly reclassified upward, and NRI was 0.10 (95% CI: 0.03–0.18; *p* = 0.005) (Table 5). After adding cortical thickness to FRAX, 37 women with fracture (17.5%) were correctly reclassified upward, 25 women without fracture (10.8%) were incorrectly reclassified upward, and NRI was 0.07 (95% CI: 0.00–0.14; *p* = 0.060). After adding cortical porosity to Garvan, 35 women with fracture (16.6%) were correctly reclassified upward, 26 women without fracture (11.2%) were incorrectly reclassified upward, and NRI was 0.05 (95% CI: -02, 0.12; *p* = 0.131). After adding cortical thickness to Garvan, 29 women with fracture (13.7%) were correctly reclassified upward, 26 women without fracture (11.2%) were incorrectly reclassified upward, and NRI was 0.03 (95% CI: -0.04, 0.09; *p* = 0.451).

**Table 5:** Reclassification of women with fracture in new models after adding cortical porosity or thickness to each of the original models including FRAX or Garvan alone.

	Net reclassification improvement (NRI)				
	Event	Nonevent	Overall	95% CI	p-value
FRAX + cortical porosity^a^	0.204	-0.099	0.10	0.03, 0.18	0.005
FRAX + cortical thickness^a^	0.175	-0.108	0.07	0.00, 0.14	0.060
Garvan + cortical porosity^b^	0.166	-0.112	0.05	-0.02, 0.12	0.131
Garvan + cortical thickness^b^	0.137	-0.112	0.03	-0.04, 0.09	0.451

FRAX: Fracture Risk Assessment Tool for calculation of the 10-year probability of major fracture. Garvan: Fracture Risk estimate of the 10-year fracture risk for any fragility fracture.

^a^Compared with FRAX alone.

^b^Compared with Garvan alone.

## Discussion

We reported that women with fracture had higher cortical porosity, thinner cortices, and smaller cortical area. Cortical porosity and thickness remained associated with prevalent fracture, independent of FRAX and Garvan estimates, and increased the AUC. Measurement of cortical porosity and thickness identified additional women with fracture than those identified using FRAX and Garvan alone. Moreover, cortical porosity improved net reclassification of women with fracture compared with FRAX alone.

The development of FRAX and Garvan tools have improved the fracture risk prediction compared to the use of aBMD alone, and both tools are well validated [6,14,27–29]. However, both tools have limitations–specifically, the omission of other risk factors that are not included in FRAX and Garvan that can influence fracture risk and potentially enhance the predictive ability of these tools. For example, TBS, a measurement derived from lumbar spine DXA images, is a well-documented risk factor for fracture, independent of aBMD and FRAX, in many cross-sectional and prospective studies, and can be included in the FRAX estimate [9,10]. However, the independent contribution from TBS to fracture risk is small [10].

To our knowledge, there is only one prospective study evaluating the predictive role of cortical porosity on incident fracture [13]. Cortical area and mass, but not porosity, at the distal tibia predicted any type of incident fracture in older men, assessed using HR-pQCT [13]. The lack of association of cortical porosity with incident fracture [28] was in contrast to previous studies suggesting cortical porosity was associated with prevalent fracture [12,14–16]. As most of the cases had wrist fractures, we showed that cortical porosity of the proximal femur was associated with prevalent wrist fractures. Another recent study showed that cortical porosity of the distal tibia was associated with prevalent hip fracture [16], but these studies did not investigate how cortical porosity is associated with vertebral fracture. As bone fragility is a general condition, we assume that cortical porosity, at any site, may be associated with any type of fracture.

Our group previously reported that sensitivity for fracture improved when cortical porosity was combined with FRAX, but over 50% of fracture cases were still unidentified from either of those measures [14]. In this study, we further explored whether inclusion of additional cortical parameters, such as cortical thickness and area, could provide additional information about fracture risk beyond the existing tools. Both cortical porosity and thickness were associated with fracture risk independent of aBMD, FRAX, and Garvan, and slightly increased the AUC. The sensitivity also increased and specificity remained high. However, when combing cortical porosity and thickness in the same model with FRAX and Garvan independently, cortical thickness was no longer associated with fracture, independent of cortical porosity. Moreover, about half of the fracture cases remained unidentified when these cortical parameters were added to Garvan or FRAX estimates. These results suggest that cortical porosity may be the most important cortical parameter and a potential predictor of fracture risk. The contribution from cortical thickness or cortical area to fracture risk seems to be modest. Further prospective studies are needed to determine whether cortical parameters provide independent information regarding fracture risk beyond FRAX and Garvan tools. Assessment of cortical porosity may be particularly of interest to identify the fracture risk in individuals without osteoporosis [14], and in those without a high Garvan or FRAX estimate.

In order to improve the sensitivity and still achieve high specificity, we explored the tradeoff for FRAX and Garvan at selected thresholds above 15%, 20%, and 25%, respectively. For FRAX, we considered a threshold >20% as the best cutoff; although the sensitivity was 25%, the specificity was 94%. When using a Garvan threshold >20%, the sensitivity was 47% (which agreed with a previous report [28]) and specificity was 82%. However, we wanted a threshold with better specificity (at least 85–90%) for each of the traits considered for further analysis in order to minimize the number of false positives. Using a Garvan threshold >25% was therefore considered as an optimal cutoff in the current data, and although the sensitivity was 35% the specificity was 92%. Combinations of risk factors increased sensitivity and maintained high specificity; however, some specificity was lost.

Prior fractures are included in both the FRAX and Garvan tools. However, while Garvan includes the number of prior fractures, FRAX does not. This may capture additional risk and contribute to differences in the performance between these tools. Another possible explanation of differences could be that the fall history is included in Garvan, but not in FRAX [7,10,28]. However, only about 5% of falls in the elderly result in a fracture [30,31]. In this study, those with one or more falls had no higher risk for fracture than those without. Secondary osteoporosis due to chronic diseases or early menopause are well-known risk factors for fracture and are included in FRAX [5,6]. However, the individual FRAX estimate remained unchanged after inclusion of secondary osteoporosis because the risk of fracture related to secondary osteoporosis is captured by aBMD [5]. Although FRAX and Garvan tools were designed to predict incident fracture prospectively, we believe it is useful to evaluate associations in retrospective settings, as it may provide interesting suggestions on risk factors that could be important to study in future prospective studies [12,32]. Ideally, we should have included vertebral fractures and more hip fractures. However, most of those with hip fracture had metal in the hip region and could not be included as metal makes noise in the CT images at both sides. Additionally, most of the patients suffering a vertebral fracture were not admitted to the hospital for an x-ray verification of fracture. The inclusion of largely wrist and humerus fractures are still of interest because these are typical osteoporotic fractures [12,29].

The strength of this nested case-control study was that it was based on a general population, fractures were x-ray verified, and cortical parameters were quantified at the proximal femur, a central site, and a common site for the most serious fragility fracture. The benefit and novelty of using the non-threshold-based software was how it was different from traditional porosity measurements. Porosity was presented here as a void fraction, and not a visually quantifiable estimate based on size and dimension. Our measure was more inclusive by encompassing porosity of both the compact cortex and TZ, and by taking into account the partial volume effect. As a result, the values of porosity were higher [12,14,18,19,24,25,33] than previously reports using other methods [13,15–16]. Studies using traditional methods to quantify porosity presented ranges from 1% to 15% likely due to only quantifying porosity of the compact cortex and porosity of completely empty voxels [13,15–16]; thus, this threshold-based image analysis underestimates porosity [24,33].

This study had several limitations. The retrospective case-control design may have introduced selection bias. The index fracture occurred at a median of 6.6 y before the women had their measurements performed. In addition, most of the women with hip fracture could not be included as metal can generate noise in the CT images. Lastly, the subtrochanteric region contained little trabecular bone, so its contribution to fracture risk could not be evaluated in this data.

In conclusion, cortical porosity and thickness were associated with increased odds for fracture, independent of aBMD, FRAX, and Garvan estimates, and slightly improved the AUC. Adding cortical porosity to existing tools may be helpful to improve fracture risk assessment beyond existing FRAX and Garvan tools, and help to identify those patients who will benefit from treatment. Further prospective studies are needed to determine whether cortical porosity or other bone traits predict fracture. Moreover, scanning procedures with low radiation, low cost, and low demand on the facilities offering these measurements need to be developed.
